# Combined Use of Gene Expression Modeling and siRNA Screening Identifies Genes and Pathways Which Enhance the Activity of Cisplatin When Added at No Effect Levels to Non-Small Cell Lung Cancer Cells *In Vitro*

**DOI:** 10.1371/journal.pone.0150675

**Published:** 2016-03-03

**Authors:** Ada W. Y. Leung, Stacy S. Hung, Ian Backstrom, Daniel Ricaurte, Brian Kwok, Steven Poon, Steven McKinney, Romulo Segovia, Jenna Rawji, Mohammed A. Qadir, Samuel Aparicio, Peter C. Stirling, Christian Steidl, Marcel B. Bally

**Affiliations:** 1 Experimental Therapeutics, BC Cancer Research Centre, Vancouver, BC, Canada; 2 Department of Pathology and Laboratory Medicine, University of British Columbia, Vancouver, BC, Canada; 3 Centre for Lymphoid Cancers, BC Cancer Agency, Vancouver, BC, Canada; 4 Molecular Oncology, BC Cancer Research Centre, Vancouver, BC, Canada; 5 Terry Fox Laboratory, BC Cancer Agency, Vancouver, BC, Canada; 6 Faculty of Pharmaceutical Sciences, University of British Columbia, Vancouver, BC, Canada; 7 Centre for Drug Research and Development, Vancouver, BC, Canada; University of South Alabama Mitchell Cancer Institute, UNITED STATES

## Abstract

Platinum-based combination chemotherapy is the standard treatment for advanced non-small cell lung cancer (NSCLC). While cisplatin is effective, its use is not curative and resistance often emerges. As a consequence of microenvironmental heterogeneity, many tumour cells are exposed to sub-lethal doses of cisplatin. Further, genomic heterogeneity and unique tumor cell sub-populations with reduced sensitivities to cisplatin play a role in its effectiveness within a site of tumor growth. Being exposed to sub-lethal doses will induce changes in gene expression that contribute to the tumour cell’s ability to survive and eventually contribute to the selective pressures leading to cisplatin resistance. Such changes in gene expression, therefore, may contribute to cytoprotective mechanisms. Here, we report on studies designed to uncover how tumour cells respond to sub-lethal doses of cisplatin. A microarray study revealed changes in gene expressions that occurred when A549 cells were exposed to a no-observed-effect level (NOEL) of cisplatin (e.g. the IC_10_). These data were integrated with results from a genome-wide siRNA screen looking for novel therapeutic targets that when inhibited transformed a NOEL of cisplatin into one that induced significant increases in lethality. Pathway analyses were performed to identify pathways that could be targeted to enhance cisplatin activity. We found that over 100 genes were differentially expressed when A549 cells were exposed to a NOEL of cisplatin. Pathways associated with apoptosis and DNA repair were activated. The siRNA screen revealed the importance of the hedgehog, cell cycle regulation, and insulin action pathways in A549 cell survival and response to cisplatin treatment. Results from both datasets suggest that RRM2B, CABYR, ALDH3A1, and FHL2 could be further explored as cisplatin-enhancing gene targets. Finally, pathways involved in repairing double-strand DNA breaks and INO80 chromatin remodeling were enriched in both datasets, warranting further research into combinations of cisplatin and therapeutics targeting these pathways.

## Introduction

Future approaches to increase the survival of patients with aggressive cancers must address the problem of tumor heterogeneity by remaining focused on broad spectrum drugs which already provide some meaningful therapeutic benefits. Standard-of-care drugs (e.g., cisplatin, doxorubicin, irinotecan, gemcitabine) will not be replaced in the near future because when used in combinations they produce significant improvements in overall survival [1–5]. These therapeutic benefits, however, are typically achieved when using drug doses that cause acute and chronic toxicities. Our research is attempting to define strategies that will enhance the activity of these drugs and reduce their toxicities through 1) approaches that consider how cancer cells protect themselves from the cytotoxic effects of the drugs and 2) drug delivery approaches that can ensure all drugs used in a combination are delivered to the right location and in the correct amounts to achieve optimal treatment outcomes. In cases where drug delivery is limited by the inadequate blood supply through tumor associated blood vessels as well as tissue specific barriers (e.g. blood-to-brain or stromal barriers), it is recognized that tumor cells are exposed to a gradient of drug concentrations [6]. Some regions within the tumor are exposed to lethal concentrations while others are exposed to sub-lethal levels of the drug(s). Tumor cells exposed to sub-lethal doses develop survival responses that protect them while also allowing for selection of drug resistant tumor cell subpopulations. The ability of cancer cells to adapt via intrinsic and acquired cytoprotective responses when first exposed to sub-lethal drug concentrations is one factor that limits the effectiveness of chemotherapeutic drugs.

Here, we describe studies to better understand how a chemotherapy naive non-small cell lung cancer (NSCLC) cell line responds when it was exposed to a cisplatin dose that caused less than a 10% loss in cell viability as determined in a 3-day high content screening assay. This drug dose was defined as the no-observed-effect level (NOEL). Two studies were completed and the results were combined to develop an understanding of how tumor cells respond when exposed to sub-lethal cisplatin doses and to determine whether these responses could be exploited to enhance cisplatin activity (i.e. causing a NOEL of cisplatin to become lethal). A microarray study examined changes in gene expressions in an adenocarcinoma NSCLC cell line A549 following treatment with a NOEL of cisplatin. In addition, a genome-wide siRNA screen was completed to identify genes that could be targeted to enhance the cytotoxic effects of cisplatin in these cells. The goal of this study is not only to identify genes and pathways that are over-expressed in response to a NOEL of cisplatin, but also to identify the genes and pathways within this list that when silenced, transform the NOEL of cisplatin to a lethal dose. When analyzing the two datasets, we did identify targets from the siRNA screen that could potentiate cisplatin activity but were not differentially expressed in the microarray study. Some of those targets were further explored and the results were disclosed in a previous publication [7]. In this study, we linked results highlighting genes that were overexpressed following low-dose cisplatin exposure to genes that when silenced enhanced the activity of cisplatin when added at a NOEL. Four genes (RRM2B, CABYR, ALDH3A1, and FHL2) were identified that could be further explored as cisplatin modulators. Further, the double strand DNA homologous repair and INO80 chromatin remodeling pathways were recognized as important targets for improving the effectiveness of low-dose cisplatin.

## Methods

### Study Design

**Fig 1:**
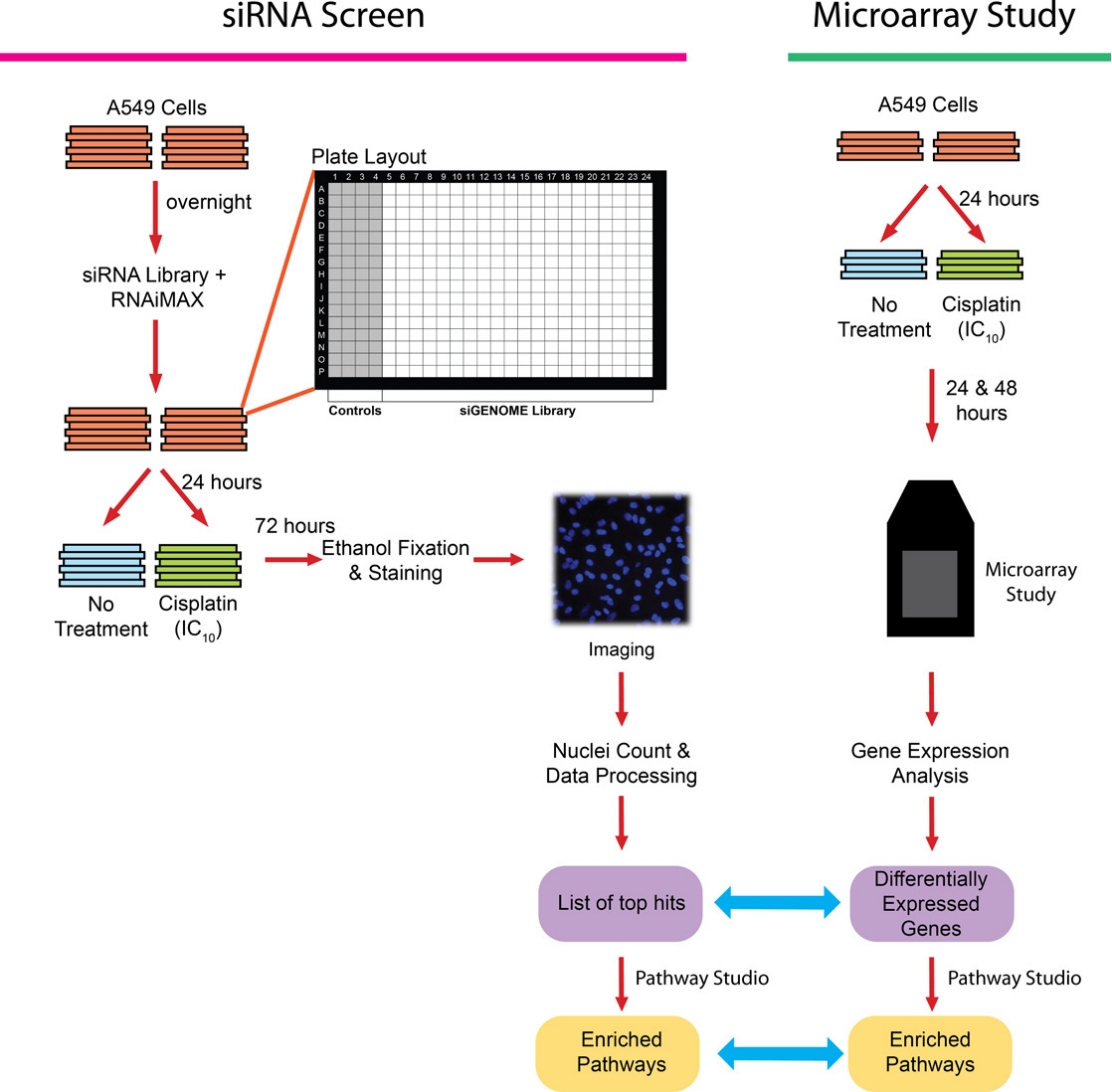
Experimental Design. The schematic diagram of the siRNA screen is illustrated on the left and the microarray gene expression study on the right. The data from the two studies were analyzed as described in the methods section. The top hits from the siRNA screen and the differentially expressed genes were compared at the genetic level to identify genes that may be targeted to enhance cisplatin activity. The two lists were separately processed through Pathway Studio and the resulting pathways were compared subsequently to determine the most critical pathways to be targeted in combination with cisplatin.

A schematic diagram of the study design is shown in Fig 1. Briefly, a high-throughput siRNA screen (see below for detailed methods) was performed to identify genes and pathways that could be inhibited to enhance the cytotoxic effects of low-dose cisplatin against A549 cells. A gene expression study was performed separately to identify differentially expressed genes and pathways in response to the same NOEL of cisplatin. The two independent studies were then compared at the gene and pathway levels.

### Cell Culture

A549 and H460 cells were obtained from the American Type Culture Collection (Manassas, VA). Cells were maintained at 37°C and 5% CO_2_ in RPMI 1640 with L-glutamine (Gibco, Grand Island, NY) supplemented with 10% fetal bovine serum (Gibco, Grand Island, NY).

### Defining the No Observable Effect Level of Cisplatin

The IC_10_ of cisplatin in a 3-day high content screen was defined in these studies as the no observed-effect level (NOEL). To establish the IC_10_ of cisplatin, cells were seeded at 2000 cells/well in a flat-bottom 384-well plate (Greiner Bio-One, Monroe, NC). Cisplatin dose response curves were determined by adding cisplatin (Mayne Pharma, Salisbury South, Australia) at various concentrations to the cells. At 72 hours post-treatment, the cells were stained for 20 minutes with dye exclusion marker Hoechst 33342 (16.2 μM, Invitrogen, Carlsbad, CA) and the nuclear stain ethidium homodimer I (EthD-I; 1μM, Biotium, Hayward, CA) and then imaged via the IN Cell Analyzer 1000 (GE Healthcare, Waukesha, WI). Nine non-overlapping images were taken per well and analyzed using the IN Cell Developer Toolbox v1.9 software (GE Healthcare, Waukesha, WI). Viable cell counts were determined by subtracting dead cell counts (EthD-I stained) from the total nuclei count (Hoechst-stained) in each well. The raw data were then normalized to untreated controls to generate a dose response curve, where a fraction affected (Fa) of 0 represents null effect from treatment and a Fa of 1 indicates 100% cell death. The dose that induced a Fa of 0.1 was the IC_10_. At this concentration, no morphological changes were observed relative to untreated controls, hence it was chosen as the NOEL for subsequent studies.

### Whole Genome siRNA (WGS) Screen

As described earlier, cells were seeded at 2000 cells/well in 384-well plates [7]. 24 hours later, the cells were transfected with a siGENOME siRNA library targeting the druggable genome (Dharmacon Technologies, Lafayette, CO). Each gene was targeted with a SMARTpool of four siRNA duplexes (to minimize off-target effects) at a total siRNA concentration of 25 nM; where 0.065 μL/well of RNAiMAX (Invitrogen) was used as a transfection reagent. Each plate had four controls randomly spotted in the first four columns of each plate to account for positioning effects (see Fig 1): RNAiMAX (transfection reagent control; 24 wells/plate), PLK1 (transfection efficiency control [8]; 16 wells/plate), scrambled siRNA (non-targeting siRNA control; 8 wells/plate), and BRCA2 targeted siRNA (a cisplatin potentiating positive control [9]; 16 wells/plate). For quality control, full plates of controls with no siRNA, +/- transfection reagent, and +/- cisplatin were screened in triplicates once per week. A cisplatin dose response curve was also generated on each cisplatin treatment day to ensure accurate drug dilution and addition. At 24 hours post-transfection, 0.551 μM cisplatin was added to the control and transfected cells or the same volume of media was added to the no-cisplatin controls. Seventy-two hours following cisplatin addition the cells were fixed with 95% ethanol and the nuclei were stained with 16.2 μM of Hoechst 33342. The plates were then imaged using the IN Cell Analyzer 1000 as described above. Total nuclei count in each well was determined using the IN Cell Developer Toolbox 1.9 software. All siRNA dilutions were carried out using a Microlab STARlet (Hamilton Robotics). Cell seeding, siRNA addition to cells, cisplatin treatment, ethanol fixation, and nuclear staining procedures were performed via Hydra DT (Thermo Fisher Scientific). The Multidrop Combi Reagent Dispenser (Thermo Fisher Scientific) was also used to facilitate the ethanol fixation process. The entire WGS was completed in 18 cycles, with each cycle being an iteration of a 6-day experiment.

### siRNA Screen Analysis

Nine non-overlapping fields were imaged in each well. Cell counts were determined by estimating the number of stained cells in each image using our cell detection algorithms [10] and the median cell counts were used to compare cell survival in untreated versus cisplatin-treated cells when a particular gene was silenced. The median was chosen over the average to account for single-well experiment failures (e.g. transfection failures) by using the values of the other two wells. A Gene Score was computed for each gene using the equation: Gene Score = (100-|Survival Index– 100|) x Potentiation Effect. The Gene Score was then used to rank the list of genes based on each gene’s similarity to the positive BRCA2 control. The Survival Index is an estimate of the lethality of gene knockdown alone and is determined based on the no-cisplatin median cell count normalized to the median cell counts from RNAiMAX transfection reagent controls. The Potentiation Effect is an estimate of the extent of cisplatin potentiation calculated by taking the Difference of the median cell counts in cisplatin-treated wells from the corresponding untreated wells and then normalizing to the difference detected from the positive control BRCA2.

For the entire screen data, a linear mixed effects model was employed to statistically account for the differences in response due to the well location of the plate (e.g. plate edge effects where lower cell counts are typically observed and reagent pipetting effects where a fixed pattern of reagent dispensing is noticed) and plate-to-plate experimental difference. Multiple comparison adjustments were performed using the Benjamini-Hochberg approach for p-values [11]. All hits presented here were considered statistically significant based on the adjusted p-values. For pathway analysis, the distributions of the Difference, Survival Index, and Gene Score values were assessed for threshold variability and a high-confidence set of non-lethal gene knockdown candidates was defined (90^th^ percentile of Difference, 75^th^ percentile of Survival Index), reducing the list of genes from 21121 to 480. These genes were then assessed for pathway enrichment.

### Microarray Analysis

Cells were seeded in 96-well plates at 5000 cells/well. On the following day, the cells were either untreated or treated with the empirically determined IC_10_ of cisplatin. At 24 and 48 hours post-treatment, total RNA was isolated using the Qiagen RNeasy plus mini kit (Qiagen, Valencia, CA). The RNA was quantified using the ND-1000 UV-VIS spectrophotometer (NanoDrop Technologies, Wilmington, DE) and RNA integrity was verified with the Agilent 2100 bioanalyzer (Agilent Technologies, Palo Alto, CA). Three independent experiments were performed and all RNA samples were sent to The Centre for Applied Genomics (TCAG) at the Hospital for Sick Children (Toronto, ON), where probe labelling and hybridization were performed according to manufacturer’s protocol (Affymetrix, Santa Clara, CA). The microarray analysis was performed using the Affymetrix GeneChip Human Exon 1.0 ST Array, which contains approximately 40 probes per gene, enabling expression analysis at both the exon level (which distinguishes between different isoforms) and at the gene level (where a single expression value summarizes data collected from all probes of the same gene). Probe cell intensity (CEL) files from TCGA were analyzed using the Affymetrix Expression Console software and Affymetrix Power Tools. For quality control, Array data was normalized using robust multi-array average (RMA) normalization [12]. Density and boxplot distributions of signal intensities were examined for consistency across all samples before and after normalization. Multidimensional scaling (MDS) was applied: one outlier (a sample set obtained at 24h) was identified and was discarded from the analysis. Differential and alternate gene expression analyses were carried out using the Limma package in R [13] to contrast treated samples (NOEL of cisplatin) to control samples (no cisplatin). A p-value cut-off of 0.005 was used to obtain a short-list of differentially expressed genes.

### Identification of Cisplatin Enhancers from the Two Datasets

The siRNA screen parameters (Survival Index, Difference, and Gene Score) were matched for each gene within the list of differentially expressed genes from the microarray study. A Gene Score rank of <2000 was applied to narrow the list of differentially expressed genes from 151 to 10. Of these potential targets, those with favourable siRNA screen parameters suggestive of cisplatin potentiation (Survival Index and % Viability in the presence of cisplatin) were selected for further validation studies (see below). Fisher’s exact test was used to evaluate the statistical significance of the overlapped gene targets.

### Pathway Analysis

All pathway analyses were completed using Pathway Studio (Ariadne Genomics) [14] and the bioinformatics resource DAVID [15,16]. To compare the siRNA screen and the microarray data, the gene lists obtained from the two studies were analyzed separately for enriched cellular processes, cell signalling, receptor signalling, and metabolic pathways. Pathways with a p-value of <0.05 were considered significant and overlaid for comparison.

### Target Validation Studies: Quantitative RT-PCR and Clonogenic Assays

For quantitative RT-PCR, total RNA was extracted as described above. The QuantiTect Reverse Transcription Kit (Qiagen) was used to eliminate genomic DNA and to synthesize cDNA from 1 μg of total RNA. RT-qPCR was performed in triplicates via the 7900HT system (Applied Biosystems, Foster City, CA). Reactions were prepared with the 2X TaqMan fast advanced master mix and 20X Taqman gene expression assays (Table 1; Applied Biosystems) according to manufacturer’s protocol. Data were analyzed using the SDS2.2 software (Applied Biosystems) and the relative messenger RNA quantity was determined using the ddCt method with GAPDH as the endogenous control.

**Table 1:** Pathways essential for A549 cell survival and cisplatin sensitivity.

Pathway	Gene Name	Gene Knockdown (% Survival)	Gene Knockdown + Cisplatin^a^ (%Survival)	Gene Score Rank^b^
**Hedgehog Pathway**	SMARCE1	100.62	37.38	7
	MBTD1	100.15	44.16	21
	CDT1	102.14	46.16	47
	TERF2	93.17	33.54	48
	FBXL11	98.16	48.52	52
	ANAPC1	101.72	58.34	66
	UBC	12.43	41.23	18976
	GDF1	18.63	16.83	18657
	SUV39H1	27.69	30.21	18823
	UBE2M	28.31	35.51	18929
**Insulin Action**	PIK3R1	103.45	44.86	22
	DDX56	91.14	29.04	37
	RPS6KA3	96.97	41.49	41
	PRKAA2	100.68	46.67	46
	OSBPL9	106.00	44.74	50
	ELF2	106.01	54.12	98
	SLC25A11	18.13	35.16	18955
	MRPL32	22.67	21.64	18669
	RPL21	22.82	23.29	18734
	RPS18	23.35	15.90	18331
	RPL19	24.52	24.52	18715
	SLC25A14	25.17	25.84	18738
	SNX9	26.46	29.82	18839
**Cell Cycle Regulation**	MBTD1	100.15	44.16	21
	PIK3R1	103.45	44.86	22
	PIGF	93.63	40.76	35
	RPS6KA3	96.97	41.49	41
	RAN	11.38	16.90	18810
	TPX2	15.61	17.66	18761
	POLR2A	17.94	16.93	18681
	SF3B5	24.71	25.58	18747
	ESPL1	27.65	29.82	18816
	SUV39H1	27.69	30.21	18823

^**a**^The cisplatin dose used caused no observable effects (<10% loss in cell viability) under non-silencing conditions.

^**b**^**Gene Score Rank** is the ranking of genes as cisplatin potentiators based on the Gene Score (calculation described in Methods). A lower value for the rank suggests that the target when silenced enhances the cytotoxic effects of cisplatin.

For colony formation assays, cells were seeded at 200,000 cells/well in 6-well plates and then transfected with a pool of three different siRNA Stealth duplexes (Life Technologies) targeting the gene of interest using RNAiMAX. The Stealth RNAi negative control kit (Life Technologies) was used as scramble control. At 24 hours post-transfection, the cells were exposed to an empirically determined IC_10_ of cisplatin for 24 hours, harvested by trypsinization, and then seeded at 500 cells per well in triplicates. The cells were incubated at 37°C, 5% CO_2_ for 14 days without disturbance. The colonies formed were fixed with 6.25% glutaraldehyde (Sigma) and stained with 0.5% crystal violet (Sigma) for 30 minutes, washed with distilled water, dried overnight, and then counted the following day. Plating efficiency (PE) was defined as the percentage of trypan blue excluding cells that formed colonies of >50 cells (PE = [(no. of colonies formed/no. of cells seeded) x100%]). Statistical analyses were performed using two-way ANOVA followed by the Holm-Sidak test. An adjusted p-value of less than 0.05 was considered statistically significant.

### Pathway Validation in Yeasts

Yeast strains were derived from the MATa, BY4741 Yeast Knockout Collection [17]. Overnight cultures were diluted to an optical density at 600 nanometers (OD600) of approximately 0.07 in media with or without 40μM cisplatin (Sigma) and grown in a Tecan M200 plate reader for 24 hours at 30°C. Shaking and OD600 measurements were done every 30 minutes during the growth period. Growth curves were analyzed essentially as described [18,19]. Briefly, 4 to 6 replicate curves for each condition were generated and the area under each curve was expressed as a proportion of the corresponding wildtype (WT) untreated sample. Multiplying the fitness effect of cisplatin on WT cells by the fitness effect of the indicated mutation created the Expected fitness based on the product or multiplicative model [20]. The observed and expected values were compared using a T-test.

## Results and Discussion

### RNAi Screen Reveals Three Important Pathways for the Survival and Cisplatin Sensitivity of A549 Cells

**Fig 2:**
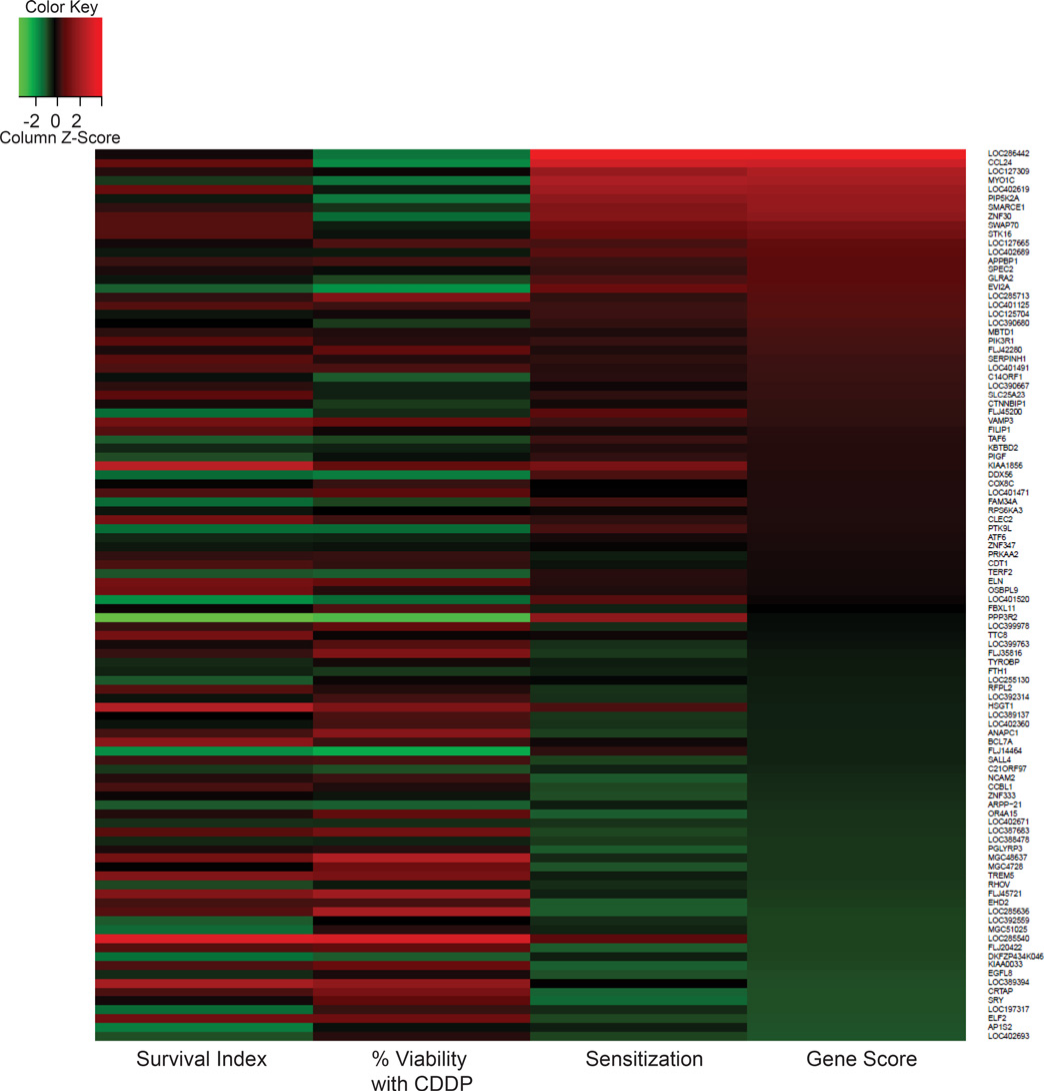
Top 100 hits from the genome-wide siRNA screen based on gene score. The survival index is a measure of cell viability relative to negative controls; % viability with CDDP represents the amount of cell death induced in the presence of both gene knockdown and cisplatin; sensitization is the difference between the survival index and % viability with CDDP; gene score takes into account both the survival index and level of cisplatin activity enhancement (see Methods).

The siRNA screen was designed to identify genes that when inhibited would sensitize NSCLC cells to cisplatin treatment. Prior to the whole genome screen (WGS), a preliminary kinome screen was conducted to establish the screening parameters, with some of the targets identified being further validated [21]. Cisplatin-potentiating targets, shown in Fig 2, were identified using the “gene score”, which considered two selection criteria: 1) gene knockdown alone had to exhibit little or no effect on cell viability (survival index) and 2) gene knockdown sensitized the cells to the cytotoxic effects of cisplatin. The survival index is a measure of the lethality of the gene knockdown alone while the sensitization or potentiation effect is a measure of the difference in cell viability in the presence and absence of low-dose (IC_10_) cisplatin. These two factors give rise to the Gene Score (see Methods) which was used to rank all potential targets.

Several other groups have previously conducted siRNA screens for cisplatin modulators. Similar to the study presented here, Bartz *et al*. performed a genome-wide siRNA screen; however, the study evaluated effects in HeLa cells. All other published studies appear to focus on the kinases (778 genes) or the druggable genome (7000 genes) [9,22–24]. While most published screens utilized metabolic assays to determine cell viability, the use of nuclei count in this study avoids inaccuracies caused by alterations in cellular metabolism due to a particular gene knockdown [25]. The high content screen used in our studies also allowed us to establish whether cisplatin treatment caused morphological changes in the cells not normally considered in metabolic assays.

This study is the first to explore the responses of NSCLC cells exposed to a NOEL of cisplatin. As we reported earlier, there is still considerable overlap between our screen and those performed by other groups despite differences in cell lines, siRNA library, cisplatin dosage, time point, and endpoint assay used [21]. For instance, BRCA1, BRCA2, RAD18, REV1L, and RFWD3 were identified in the study reported by Bartz *et al*. and these genes were also found to potentiate cisplatin activity in our screen. CALM1, NRGN and PTK9L were identified in the study reported by Arora *et al*. and STK16 was identified in the study reported by Swanton *et al*. These genes also significantly enhanced cisplatin activity in our study. Interestingly, CHEK1 was shown to enhance cisplatin sensitivity in the screens reported by Bartz and Arora’s groups; however, our results were consistent with those reported by Nijwening *et al*., which suggested that CHEK1 inhibition alone was lethal. We believe that our experimental design and the use of a gene score to select gene candidates have allowed us to generate a distinct list of gene candidates that may be targeted to enhance cisplatin sensitivity.

**Fig 3:**
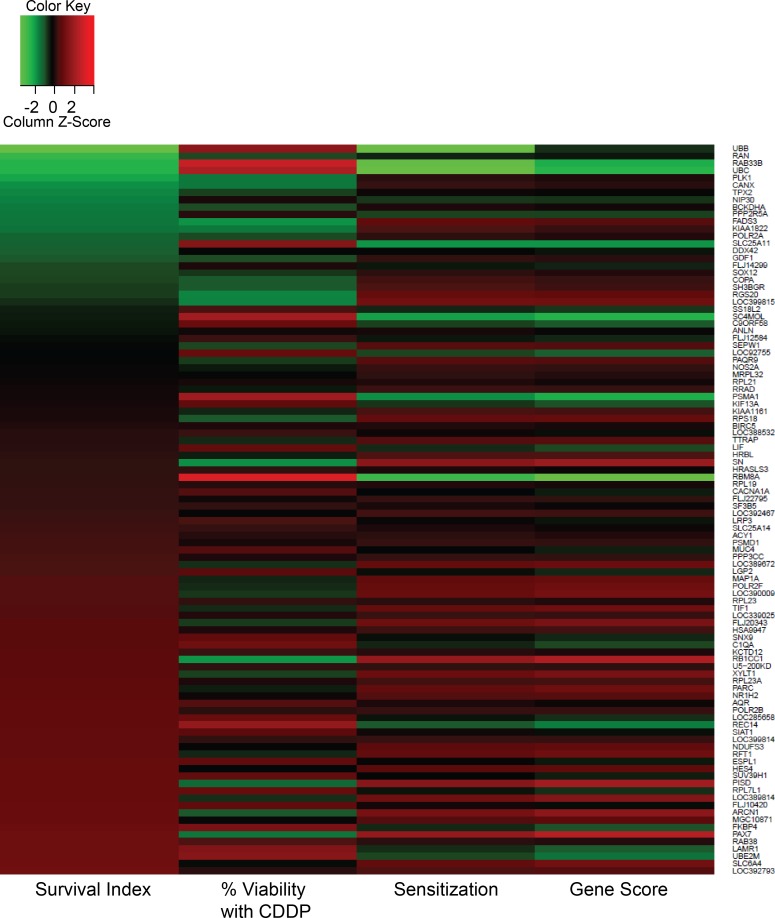
Most lethal targets identified in the siRNA screen. The 100 genes with the lowest gene score are listed; ranked by lowest to highest survival index. While a few of these genes may still sensitize A549 cells to cisplatin treatment, gene knockdown in the absence of cisplatin would cause at least 70% loss in cell viability.

The results summarized in Fig 3 identify the most lethal genes; genes that when silenced alone resulted in a low survival index, i.e. silencing resulted in at least 70% loss in cell viability in the absence of additional treatments. The top 100 cisplatin enhancers and the 100 most lethal genes were analyzed separately using Pathway Studio. The resulting pathways were compared and three pathways: Hedgehog, Insulin Action, and Cell Cycle Regulation, were found to have the most impact on A549 cell viability and cisplatin sensitivity (Table 1). The insulin action and cell cycle regulation pathways are known to be associated with cancer progression and resistance [26,27]. Activation of the insulin signaling pathway results in downstream activation of oncogenic pathways such as the phosphatidylinositol 3-kinase (PI3K) and the MAPK pathways which are important for cell growth and proliferation [28]. These pathways are also suggested to be important targets for overcoming chemoresistance [29,30]. The hedgehog pathway (Hh) is activated in multiple types of cancers including NSCLC [31–33]. This pathway is associated with cancer growth, metastasis, and drug resistance and is involved in crosstalk with other commonly deregulated pathways in cancer such as the Notch and Wnt signaling pathways [31,34]. Currently, the Hh pathway inhibitor vismodegib is approved for use in basal cell carcinoma while several other inhibitors are still being tested in clinical trials. Vismodegib and BMS-833923 are both being evaluated in combination with a platinum-containing doublet for treatment in patients with SCLC. In NSCLC, the Hh pathway is thought to be involved in epithelial-to-mesenchymal transition [35]. Furthermore, Tian *et al*. have demonstrated that the combination of vismodegib and cisplatin is more cytotoxic in NSCLC cells than either treatment alone; a result which suggests synergistic interactions [36].

### Analysis and Validation of Microarray Data

**Fig 4:**
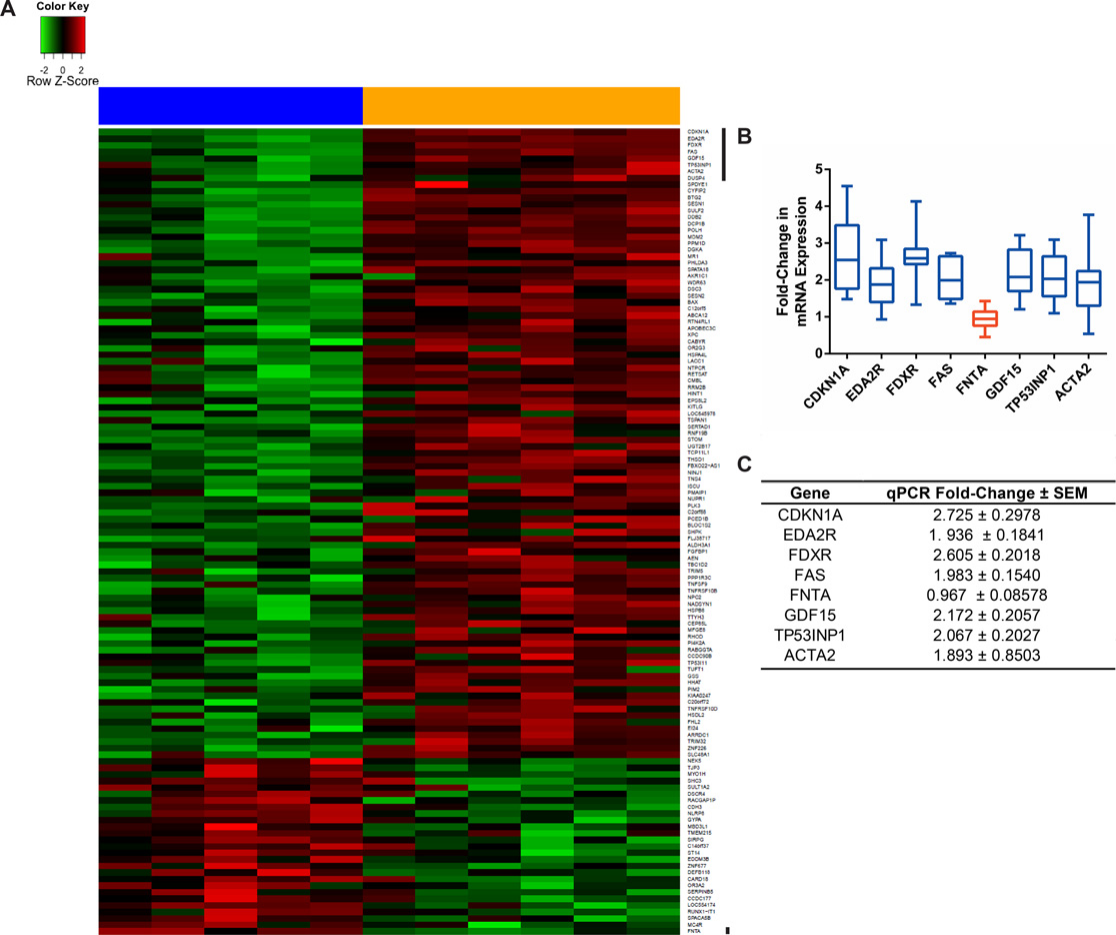
Differentially expressed genes in A549 cells following cisplatin treatment at its IC_10_. The heat map represents the gene expression profile for the differentially expressed genes identified between untreated (blue) and cisplatin-treated (orange) cells (A). Each column displays data from one microarray chip. Genes that were further validated by qPCR are highlighted with a black vertical line beside the gene. The fold-changes in mRNA expression are plotted in the box plot (B; blue = over-expression, red = under-expression after cisplatin treatment) and the averaged values are tabulated from six individual experiments at each time-point (C). All changes observed were statistically significant (p<0.05) except for FNTA.

Unlike other microarray studies completed using high drug doses (e.g. IC_50_); the microarray study presented here was completed using a NOEL of cisplatin (e.g. IC_10_). The NOEL dose was chosen to mimic a condition where cancer cells are exposed to a sub-lethal concentration of the drug and the assumption that these cells will develop adaptive response that protect them in the short term and select for resistance in the long term. These responses should be detectable at the molecular level including changes in mRNA expression. Our microarray data suggest that 151 genes were differentially expressed following exposure to the NOEL of cisplatin. Among the 151 genes, 50 were significantly down-regulated and 101 up-regulated (p<0.005) and have been summarized in Fig 4. The seven most up-regulated genes and the one most down-regulated gene were validated via quantitative RT-PCR (probes listed in S1 Table). All up-regulated genes were confirmed to be over-expressed following addition of the NOEL of cisplatin (p<0.05) while the change detected in the selected down-regulated gene was not statistically significant (Fig 4B and 4C). The log fold-change and the corresponding data from the siRNA screen for the up-regulated genes have been summarized in Table 2.

**Table 2:** Over-expressed genes from Affymetrix Exon 1.0 Array matched with siRNA Screen Data.

Gene Name	Log Fold-Change^a^	% Survival with CDDP^b^	Survival Index^c^	Potentiation^d^	Gene Score	Whole Genome Rank^e^
C2orf88	-0.3592	62.44	114.18	78.33	6722	453
**RRM2B**	**-0.4104**	**59.76**	**94.82**	**65.64**	**6224**	**706**
**CABYR**	**-0.4351**	**53.57**	**92.93**	**66.97**	**6224**	**708**
**NINJ1**	**-0.3639**	**42.73**	**85.34**	**71.76**	**6124**	**785**
**ALDH3A1**	**-0.3329**	**69.26**	**97.65**	**60.96**	**5952**	**888**
**DUSP4**	**-0.2266**	**68.01**	**109.70**	**64.92**	**5862**	**973**
TNFSF9	-0.3108	69.10	105.17	61.46	5829	1006
**FHL2**	**-0.2346**	**52.60**	**89.14**	**60.43**	**5387**	**1429**
TSPAN1	-0.4008	72.71	100.22	51.35	5124	1739
DDB2	-0.6643	62.56	93.72	49.26	4617	2587
SESN2	-0.4943	66.86	97.66	46.64	4555	2724
CCDC90B	-0.2574	74.40	103.39	46.90	4531	2763
KITLG	-0.4051	68.77	94.81	44.52	4221	3608
BAX	-0.4733	68.23	93.70	43.74	4099	3973
ARRDC1	-0.2196	68.00	94.14	41.89	3943	4504
AEN	-0.3286	67.75	94.33	41.75	3938	4525
HINT1	-0.4087	41.92	72.42	54.07	3916	4600
NPC2	-0.2917	78.04	99.92	37.23	3720	5322
PPM1D	-0.5798	72.78	93.52	39.22	3667	5564
TP53I11	-0.2545	87.94	106.40	38.59	3612	5791
TNFRSF10B	-0.3096	56.58	82.16	43.91	3608	5804
RHOD	-0.2825	80.37	97.97	36.78	3604	5822
XPC	-0.4428	72.92	95.88	36.85	3533	6141
C12orf5	-0.4646	78.23	99.59	35.28	3514	6234
MFGE8	-0.2832	52.17	78.63	44.56	3504	6278
SHPK	-0.3421	80.07	103.23	36.07	3491	6320
CYFIP2	-0.7552	84.53	105.99	36.70	3450	6510
NADSYN1	-0.2884	58.95	82.90	41.06	3404	6744
UGT2B17	-0.3809	72.83	92.62	36.74	3403	6749
MDM2	-0.5886	61.43	82.95	40.70	3376	6876
TNFRSF10D	-0.2391	37.02	65.58	51.30	3364	6937
ACTA2	-0.7726	30.92	61.29	51.68	3168	7813
HSPB8	-0.2878	52.81	78.30	39.69	3108	8122
BTG2	-0.7545	66.15	86.55	35.30	3055	8364
NTPCR	-0.4198	57.97	82.44	37.04	3054	8367
PI4K2A	-0.2796	54.92	76.84	38.30	2943	8878
TRIM5	-0.3209	59.14	82.27	35.68	2935	8907
SERTAD1	-0.3994	68.39	88.36	32.04	2830	9420
TCP11L1	-0.3789	57.80	78.43	34.87	2735	9872
PCED1B	-0.3562	46.91	70.76	38.21	2704	10006
TBC1D2	-0.3271	70.00	88.12	30.62	2698	10032
RABGGTA	-0.2780	17.62	47.69	56.30	2685	10096
NUPR1	-0.3616	34.06	60.69	43.49	2639	10286
CMBL	-0.4166	53.44	72.52	35.72	2590	10505
EDA2R	-1.0228	65.80	83.91	30.87	2590	10508
FGFBP1	-0.3289	31.41	57.59	41.40	2384	11382
GSS	-0.2515	41.98	62.37	38.10	2376	11422
ISCU	-0.3629	41.17	62.38	37.18	2319	11659
FDXR	-0.9802	56.96	71.61	31.47	2254	11942
DGKA	-0.5798	49.71	70.06	31.68	2220	12083
C20orf72	-0.2415	70.29	86.02	25.20	2168	12317
PHLDA3	-0.5223	59.41	75.74	27.78	2104	12608
TNS4	-0.3638	32.81	56.39	36.39	2052	12825
SESN1	-0.7338	83.88	96.92	20.92	2027	12919
KIAA0247	-0.2461	74.13	86.15	23.19	1998	13047
TTYH3	-0.2870	28.18	50.94	38.90	1982	13113
POLH	-0.6136	38.26	56.83	34.69	1971	13155
HHAT	-0.2507	61.36	76.39	25.40	1940	13292
RNF19B	-0.3950	71.21	85.59	21.83	1869	13579
HSPA4L	-0.4323	53.17	69.28	25.47	1764	13984
RETSAT	-0.4176	46.47	62.79	27.98	1757	14013
EI24	-0.2226	66.46	77.29	22.64	1750	14048
STOM	-0.3936	37.41	55.85	29.58	1652	14413
WDR63	-0.5033	38.08	56.45	27.88	1574	14710
SULF2	-0.7130	57.93	69.61	21.88	1523	14901
THSD1	-0.3759	65.18	76.60	19.29	1478	15079
AKR1C1	-0.5096	49.53	62.18	23.63	1469	15107
DSC3	-0.4999	46.02	61.19	23.99	1468	15111
TP53INP1	-0.8497	42.09	54.77	26.50	1451	15161
EPS8L2	-0.4071	20.11	41.02	32.84	1347	15544
TUFT1	-0.2533	26.18	42.84	30.92	1325	15617
ABCA12	-0.4541	46.15	57.56	21.95	1263	15791
APOBEC3C	-0.4432	60.06	68.74	18.14	1247	15854
SLC48A1	-0.2010	39.53	52.25	21.50	1124	16277
MR1	-0.5546	32.07	46.61	24.04	1120	16287
PLK3	-0.3604	20.62	38.04	27.12	1032	16570
SPDYE1	-0.7914	46.79	54.66	18.35	1003	16651
CEP85L	-0.2833	62.93	69.92	14.10	986	16703
CDKN1A	-1.1354	87.25	93.56	9.81	918	16898
RTN4RL1	-0.4497	53.24	60.96	13.86	845	17105
PIM2	-0.2468	32.77	42.94	17.77	763	17314
PPP1R3C	-0.3134	36.28	43.26	13.21	572	17768
ZNF226	-0.2011	39.15	45.85	12.44	570	17773
FAS	-0.9781	33.66	39.88	12.99	518	17888
PMAIP1	-0.3623	63.15	67.00	6.55	439	18065
HSDL2	-0.2368	45.87	50.81	7.47	380	18176
GDF15	-0.8717	36.87	42.01	8.79	369	18193
TRIM32	-0.2046	30.69	34.49	7.19	248	18403

^**a**^ **Log-fold change** is the change in gene expression following cisplatin treatment in the microarray study. All other data are collected from the siRNA screen for each corresponding gene.

^**b**^ **% Survival with CDDP** is the cell viability of target-silenced cells treated with cisplatin; presented as percentage relative to non-silencing and no cisplatin controls. The dose of cisplatin used caused no observable effect (<10% loss in cell viability) under non-silencing conditions.

^**c**^ **Survival Index** is the cell viability following gene knockdown, presented as percentage relative to untreated controls

^**d**^ **Potentiation** is the difference in cell count between untreated and cisplatin-treated when the gene is silenced; presented as percentage normalized to BRCA2 positive controls.

^**e**^ **Whole Genome Rank** is the ranking of genes based on gene score.

### *RRM2B* and *CABYR* Silencing Sensitized A549 and H460 Cells to Cisplatin Treatment

**Fig 5:**
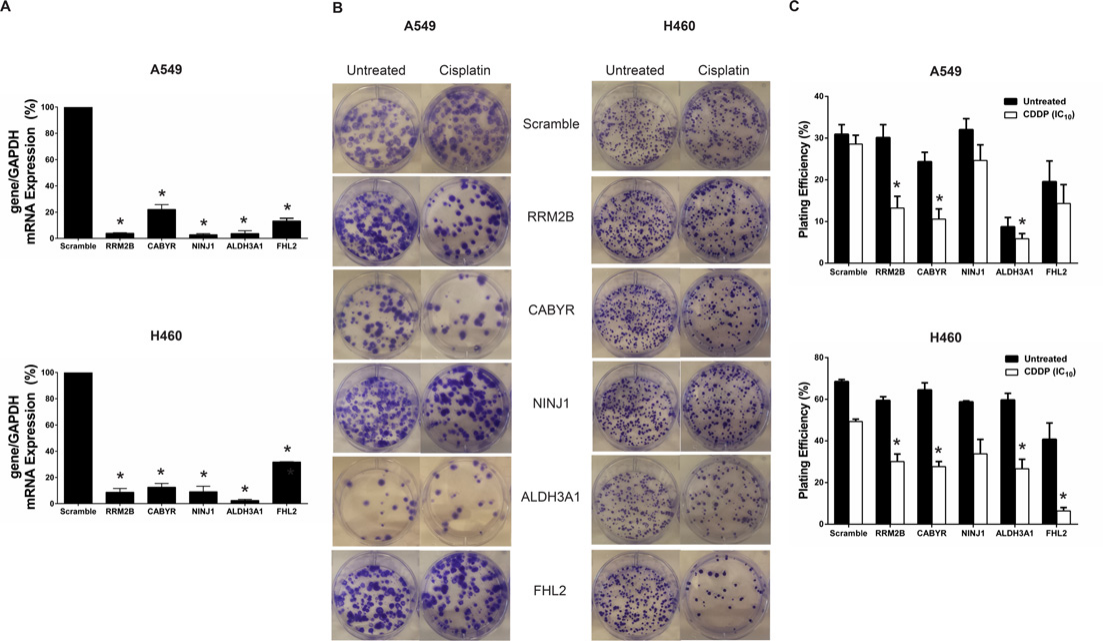
Validation studies assessing the effects of ALDH3A1, RRM2B, CABYR, FHL2, and NINJ1 silencing on cisplatin activity in A549 and H460 cell lines. Knockdown of each candidate target was confirmed via qPCR (A; *p<0.05 relative to scramble control). The representative image for each tested condition is displayed in B. The plating efficiency of each condition is plotted in separate graphs for each cell line (C; data shown as mean ± SEM; n = 3; *p<0.05).

It was anticipated that results from the siRNA screen would complement results from the gene expression studies, leading to the identification of pathways/targets that are up-regulated when cells are exposed to the NOEL of cisplatin and, when silenced, cause the NOEL to become lethal. To identify such cisplatin activity enhancers, over-expressed genes from the microarray study that were also ranked within the top 10% of genes in the siRNA screen were identified. Five of these genes (ALDH3A1, RRM2B, CABYR, FHL2, and NINJ1) were selected for further validation based on evidence of their role in cancer (Table 3). A clonogenic assay was used for target validation; an assay that assessed the long-term effects of the gene knockdown in the presence and absence of an empirically derived NOEL of cisplatin. It is important to note that the clonogenic assay assesses tumor cell survival over 14 days in contrast to the high content screen which evaluated effects after 3 days. The siRNA sequences used to suppress the expression of the selected genes for the validation studies are shown in Table 4. These siRNA sequences differed from those used in the siRNA screen to ensure that the effects observed were not sequence-specific. The level of gene knockdown was verified via qRT-PCR (Fig 5A) and the validation studies were completed in two chemotherapy-naïve NSCLC cell lines (A549 and H460) as the main objective is to improve cisplatin activity in the first-line setting [37]. The cell lines chosen are KRAS mutants and harbour wild-type p53. The results, summarized in Fig 5, illustrated four main points. First, the cisplatin-potentiating effects associated with gene silencing could be subtype or cell context-specific. For example, ALDH3A1 knockdown was lethal in A549 cells (Fig 5B), causing over 70% reduction in plating efficiency (PE) relative to the scramble control while in H460 cells, ALDH3A1 knockdown only reduced PE by 12%. In H460 cells, ALDH3A1 silencing in combination with cisplatin resulted in a 46% reduction in PE relative to scrambled cells treated with cisplatin (Fig 5C). A smaller, albeit significant, decrease in PE was also observed in the ALDH3A1-silenced A549 cells treated with cisplatin. Since ALDH3A1 is the only target for which a small molecule inhibitor (CB29) is commercially available, we attempted to validate the target by adding the maximal non-toxic dose of CB29 in combination with different concentrations of cisplatin. However, due to solubility issues and the need of using DMSO, which is known to inactivate cisplatin and other platinum-based therapeutics, the validation studies could not be completed in a meaningful way [38]. Second, while most screening approaches rely on short-term (3-day) assays, target validation should be conducted using long-term assays such as the clonogenic assay. As an example, FHL2 knockdown sensitized both A549 and H460 cells to a NOEL of cisplatin, reducing the PE by 49.8% and 87.1%, respectively. However, the gene knockdown alone caused approximately 40% loss in viability in both cell lines, which was only observed when long-term viability was assessed using the colony formation assay. Third, although sensitization to cisplatin was observed when NINJ1 was silenced in both cell lines, the effects were not statistically significant, highlighting the need for validation studies. Fourth, both RRM2B and CABYR knockdown in the cell lines appeared well-tolerated (< 25% loss in clonogenicity; Fig 5C) and in the presence of the NOEL of cisplatin, the PE was further reduced by approximately 40% relative to the scramble control (Fig 5C). In summary, RRM2B and CABYR silencing enhanced cisplatin activity in A549 cells while inhibition of four of the five selected genes sensitized H460 cells to cisplatin treatment. A study conducted on 39 cervical cancer tumour samples revealed that RRM2B is consistently up-regulated in response to chemoradiation where cisplatin was used as a radiosensitizer, suggesting that RRM2B may be a clinically relevant target for chemosensitization [39]. On the other hand, it has recently been shown that CABYR suppression enhances the cytotoxic effects of cisplatin in A549 and H460 [40]; consistent with our findings. Interestingly, CABYR is not normally expressed in the lung but has been detected in lung cancer tissues from patients [41,42]. It is therefore a clinically relevant and promising target. To determine whether CABYR knockdown only sensitized lung cancer cells to low-dose cisplatin, we performed additional clonogenic assays using a range of cisplatin concentrations (S1 Fig). Our results suggest that suppressing CABYR expression would enhance cisplatin activity significantly at low as well as high doses of cisplatin. To determine whether RRM2B and CABYR should be further pursued as therapeutic targets, future validation work could expand to additional cell lines with different genetic background or driver mutations as well as efficacy assessments *in vivo*.

**Table 3:** Potential cisplatin activity enhancers selected from the siRNA screen and microarray gene expression studies and their known roles in cancer biology.

Gene Symbol	Gene Name	Relevance to Cancer	Reference
**RRM2B**	Ribonucleotide reductase M2 B	P53-inducible subunit of the human ribonucleotide reductase important for DNA repair; Role in NSCLC unknown but controversial in other cancer types:	[43,44]
**CABYR**	Calcium binding tyrosine phosphorylation regulated protein	Cancer-testis antigen; gene expressed in lung cancer tissues and patient sera; also shown to sensitize A549 and H460 cells to cisplatin and taxol treatments *in vitro* and *in vivo*	[41,42]
**NINJ1**	Nerve injury-induced protein 1	Adhesion molecule important for nerve regeneration; overexpressed in human cancer and induced by DNA damage in a p53-dependent manner	[45]
**ALDH3A1**	Aldehyde dehydrogenase 3 family, member A1	Aldehyde dehydrogenase involved in metabolism of xenobiotics; over-expressed in NSCLC; high expression confers resistance to nitrogen mustards	[46–49]
**FHL2**	Four and a half LIM domains 2	Interact with transcription factors and proteins involved in cancer development; de-regulated in various tumour tissues	[50]

**Table 4:** siRNA Sequences Used for Target Validation.

Gene ID	Stealth siRNA Sequences (5’ to 3’)	Sequence ID
RRM2B	GCA GUU AUG GCA GAA ACC ACA GAU A	HSS121295
	GCC UGA UGU UCC AAU ACU UAG UAA A	HSS121296
	CAU UGA GUU UGU AGC UGA CAG AUU A	HSS181703
CABYR	CAG AAG GAA CGA CAC CAC AGA AGA A	HSS146764
	ACA GAC ACA GAC GAG GAC AAU GUA A	HSS146765
	GGU GAC AAA UGU GCU CCC UUU GGA A	HSS178124
NINJ1	ACG GGC CCA UCA ACG UGA ACC AUU A	HSS107188
	GCC UGG UGU UCA UCA UCG UGG UAG U	HSS107190
	GGG UGC UGC UCA UCU UCC UUG UCA A	HSS181529
ALDH3A1	GCC AAC GAU GUC AUC GUC CAC AUC A	HSS100373
	AGG AGA GGU UCG ACC AUA UCC UGU A	HSS100374
	CAG AAC CAA AUU GUG GAG AAG CUC A	HSS176687
DUSP4	CAA ACC ACU UUG AAG GAC ACU AUC A	HSS176265
	CCU GGU UCA UGG AAG CCA UAG AGU A	HSS176266
	CCC ACC UCG CAG UUC GUC UUC AGC U	HSS176267
FHL2	GCC UGA ACU GCU UCU GUG ACU UGU A	HSS142018
	CCU GCU AUG AGA AAC AAC AUG CCA U	HSS142019
	CCC UGG CAC AAG GAG UGC UUC GUG U	HSS142020

### The INO80 Chromatin Remodeling and the Double Strand DNA Homologous Repair Pathways Are Induced by Low-Dose Cisplatin and May Be Targeted to Improve Response to Platinum Treatments

To systematically compare the siRNA screen and the Affymetrix study at the pathway level, the siRNA screen hits were filtered by potentiation effect and survival index (see Methods). This resulted in a list of 480 genes that enriched for pathways involved with chromatin remodeling and DNA modification (Table 5). Similar to other microarray studies, most of the differentially expressed genes we identified were up-regulated [51,52] and were primarily involved in apoptosis induction and various types of DNA repair (Table 6). These results were further confirmed via GO analysis of the up-regulated genes (S2 Table) and are not unexpected since cisplatin is a DNA damaging agent. DNA repair must be effective and efficient for the cells to survive exposure to the agent, even when the exposure dose is low. Importantly, hierarchical clustering revealed several groups of up-regulated genes enriched in p53 signalling (p = 2 x 10^−6^), six of which are amongst the seven most over-expressed genes (S2 Fig). This finding is consistent with another study that demonstrated activation of the p53 signaling pathway in ovarian cancer in response to cisplatin treatment [53].

**Table 5:** Pathway enrichment analysis of cisplatin-potentiating gene targets from the siRNA screen.

Pathway	Associated genes	p-value^a^
INO80 Chromatin Remodeling	INO80D, DCLRE1C, SMARCD3, BAZ1A, KAT2A, KAT6A, HDAC9, CDT1, PMS2, ORC1, RAD18, CHTF8, H2AFV, NAP1L2, RFC3, MBTD1	0.0015169
Histone and DNA Methylation	SMARCD3, BAZ1A, KAT2A, KAT6A, HDAC9, SALL1, CDT1, ORC1, CHTF8, H2AFV, NAP1L2, RFC3, MBTD1	0.0032743
Histone Acetylation	SMARCD3, KPNA2, BAZ1A, KAT2A, IPO5, SORBS2, KAT6A, HDAC9, SALL1, H2AFV, NAP1L2, MBTD1	0.0049853
Hedgehog Pathway	IHH, DCLRE1C, SMARCD3, CUL4A, POT1, ANAPC1, BAZ1A, KAT2A, KAT6A, HDAC9, CDT1, PMS2, ORC1, RAD18, CHTF8, H2AFV, NAP1L2, WNT16, RFC3, KDM2A, MBTD1	0.0092622
TRRAP/TIP60 Chromatin Remodeling	Gas41, DCLRE1C, SMARCD3, BAZ1A, KAT2A, KAT6A, HDAC9, PMS2, RAD18, H2AFV, NAP1L2, HELQ, MBTD1	0.01193
Histone Ubiquitination	SMARCD3, BAZ1A, KAT2A, KAT6A, NAE1, HDAC9, CTR9, H2AFV, NAP1L2, UBE2G1, MBTD1	0.0131073
Sister Chromatid Cohesion	ANAPC1, CDT1, DLGAP5, ORC1, CHTF8, RFC3, TACC2	0.0160821
Alternative Complement Pathway	C8G, CR2, CFP	0.0236879
Double Strand DNA Homologous Repair	DCLRE1C, CDT1, PMS2, ORC1, RAD18, CHTF8, RFC3	0.0252954
AGER -> CREB/SP1 signaling	RPS6KA3, PIK3R1, S100A1	0.027402
Co-translational ER Protein Import	RPS6KA3, P4HB, HSPH1, SEC63, RPS6KA6, RPS6KC1, RPS28, NACA2, TMX3, MRPL51	0.0327461
SRCAP Chromatin Remodeling	Gas41, SMARCD3, BAZ1A, KAT2A, KAT6A, HDAC9, H2AFV, NAP1L2, MBTD1	0.0351599
Histone Sumoylation	SMARCD3, BAZ1A, KAT2A, KAT6A, HDAC9, H2AFV, NAP1L2, MBTD1	0.0446793
Metabolism of triacylglycerols	PNLIPRP3, AGPAT9, DAK	0.0474868

^**a**^The p-value is derived from the Pathway Studio software that determines the likelihood of the pathway being enriched from a random gene list of the same size.

**Table 6:** Pathway enrichment analysis of differentially expressed genes following low-dose cisplatin treatment.

Pathway	Associated genes	p-value^a^
Apoptosis	MDM2, FAS, BAX, TNFRSF10B, TNFRSF10D	0.000478709
Apoptosis Regulation	XPC, MDM2, FAS, TNFRSF10B, TNFRSF10D, TNFSF9, RRM2B, DDB2, EDA2R	0.00220469
Double Strand DNA Homologous Repair	XPC, RRM2B, POLH, DDB2	0.00406061
Single-Strand Base Excision DNA Repair	XPC, RRM2B, DDB2	0.00946967
Single-Strand Mismatch DNA Repair	XPC, RRM2B, DDB2	0.0103586
Direct DNA Repair	XPC, RRM2B, DDB2	0.0115367
Histone Phosphorylation	XPC, RRM2B, DDB2, PAK3	0.0120578
Double Strand DNA Non-Homologous Repair	XPC, RRM2B, DDB2	0.0133127
TNFR -> NF-kB signaling	FAS, TNFRSF10B	0.0151032
TNFR -> AP-1/ATF/TP53 signaling	FAS, TNFRSF10B	0.0151032
TNFR -> CREB/ELK-SRF signaling	FAS, TNFRSF10B	0.0189529
Cell Cycle Regulation	XPC, MDM2, FAS, CDKN1A, KITLG, TNFRSF10B, TNFRSF10D, TP53INP1, TNFSF9, RRM2B, TNS4, POLH, DDB2, DUSP4, PAK3, EDA2R, MBD3L1	0.0190788
INO80 Chromatin Remodeling	XPC, RRM2B, POLH, DDB2, MBD3L1	0.0211387
KIT -> STAT signaling	KITLG	0.029861
Sialophorin -> CTNNB/MYC/TP53 signaling	MDM2	0.029861
Sulfur metabolism	SULT1C3, SULT1A2	0.0332819
TNFRSF6 -> FOXO3A signaling	FAS	0.0347648
Metabolism of glucocorticoids and mineralcorticoids	SULT1C3, AKR1C1	0.0379581
Adipocytokine Signaling	FAS, TNFRSF10B, UGT2B17, TNFRSF10D, TNFSF9, DUSP4, ALDH3A1, EDA2R	0.0401247
EctodysplasinR -> LEF1 signaling	EDA2R	0.0493525

^**a**^The p-value is derived from the Pathway Studio software that determines the likelihood of the pathway being enriched from a random gene list of the same size.

**Fig 6:**
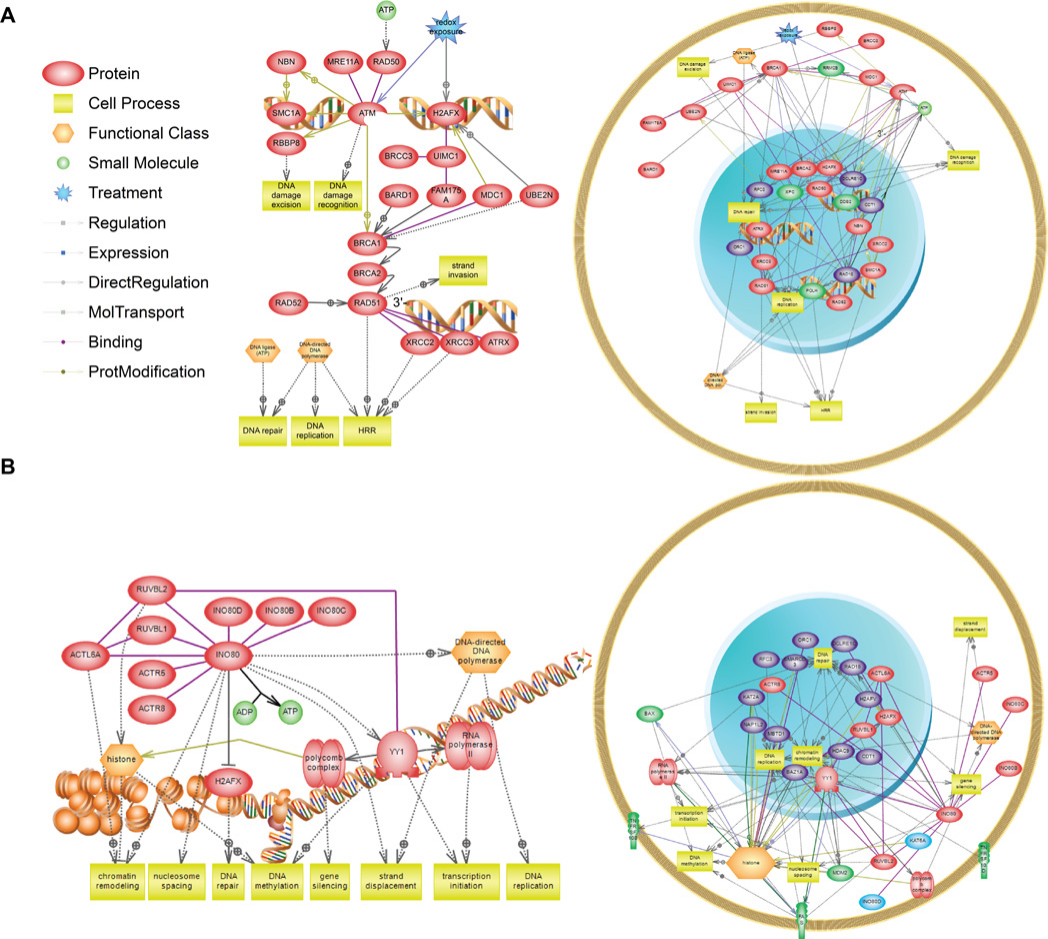
Pathways enriched in both the siRNA screen top hits and the microarray differentially expressed genes. Of the siRNA screen hits and the microarray gene list, the double stranded homologous repair (A) and the INO80 chromatin remodelling (B) pathways were enriched in both datasets. The schematic diagram of the two pathways is displayed on the left while genes from the siRNA screen (purple) and the microarray study (green) that map to these pathways are illustrated based on cellular localization (right).

A comparison of significant pathways (p-value < 0.05) from the siRNA screen and microarray study revealed enrichment in the INO80 chromatin remodeling pathway and the double strand DNA homologous repair pathway. Fig 6 illustrates the two pathways enriched in both the siRNA screen and the microarray; where hits identified from the siRNA screen are highlighted in purple and differentially expressed genes are shown in green. While ATP-dependent chromatin remodeling is known to be important for gene transcription, previous studies have also demonstrated that the INO80 chromatin remodeling complex (Fig 6B) interacts with γ-H2AX in the presence of DNA damage and is directly involved in the repair of double-stranded DNA breaks [54,55]. A working homologous recombination repair pathway plays a role in repairing cisplatin-induced DNA damage [56,57]; however, when defective, the pathway has an increased sensitivity to DNA damaging agents [58]. It is only in recent years that chromatin remodeling complexes gained recognition as being important for repairing double-stranded breaks. Our findings further suggest that the INO80 chromatin remodeling pathway is induced in response to cisplatin and may be targeted to enhance tumor cells’ sensitivity to cisplatin [59].

**Fig 7:**
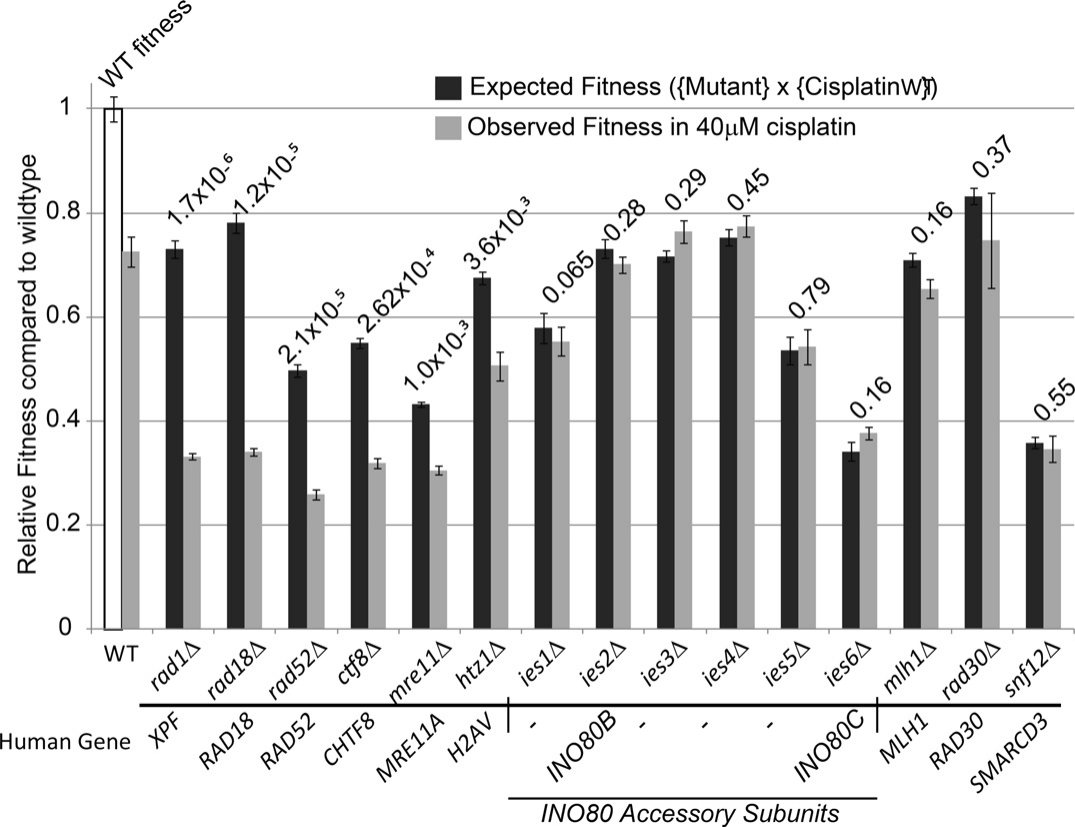
Fitness measurements for cisplatin treated yeast mutant strains. WT fitness (white bar) was used to predict the effects of cisplatin on mutant strains assuming a simple multiplication of the mutant fitness defect and the effect of cisplatin on WT. Significantly lower fitness values were observed for strains deleted for RAD1, RAD18, RAD52, CTF8, MRE11 or HTZ1 (Holm-Bonferroni corrected p-value <0.05) but not for other mutants tested. Raw p-values are indicated above each comparison. Human gene orthologues of each yeast mutant are indicated below. Errors bars are standard error of the mean.

In attempt to validate some of the cisplatin-potentiating pathways uncovered by our studies we turned to the simple model organism *Saccharomyces cerevisiae* (yeast). As expected, yeast deficient in homologous recombination (*rad52∆*) or nucleotide excision repair (*rad1∆*) were highly sensitive to cisplatin (Fig 7). In addition, RAD18, CHTF8 and H2AV genes predicted by our siRNA screen were also recapitulated as cisplatin hypersensitive when the corresponding yeast gene deletion was tested. Interestingly, deletion of yeast INO80 accessory subunits *IES1-6* did not cause cisplatin sensitivity in yeast. The yeast and human INO80 complexes have different subunit compositions and the INO80D gene, which was identified in our siRNA screen (Table 5), is not conserved in yeast. It is possible that the catalytic activity of INO80A is required to modulate cisplatin treatment response or that INO80D has a specific role in the response of human cells to cisplatin. Here, we have elucidated some components of the double-stranded homologous DNA repair and the INO80 chromatin remodeling pathways in a simplified eukaryotic system. To further understand the importance of the INO80 chromatin remodeling complex in modulating cisplatin treatment response, future studies will require testing each human subunit and performing functional assays to identify the subunit(s) that could be targeted to induce selective killing of cancer cells in the presence of low-dose cisplatin.

As mentioned above, many of the differentially expressed genes identified are involved in DNA repair. Although the nucleotide excision repair (NER) pathway is known to be a major DNA repair pathway involved in removing CDDP-DNA adducts, it was not identified as a hit in the current study primarily because of the lack of overexpression in NER genes in response to low-dose CDDP [60–63]. A previous study in melanoma has also demonstrated that majority of NER genes are not upregulated at 6 and 24 hours following CDDP treatment but CDDP resistance was still observed as the cancer cells had a higher basal level of NER gene expression than normal cells [64]. The importance of NER in CDDP treatment response is still evident in our siRNA screen, however, as about 1/3 of the NER genes in our screen were found to sensitize A549 cells to CDDP when silenced via siRNA (S3 Table), consistent with other studies in the literature [25,60,65,66]. Therefore, while we have identified two lead pathways based on both the siRNA screen and the microarray study, pathways identified from either study alone could also be further explored as cisplatin-sensitizers.

## Conclusions

The primary objective of the siRNA screen and the microarray study was to identify genes that are up-regulated in response to a NOEL of cisplatin, which if inhibited, would enhance the cytotoxic effects of the drug. Aside from genetic heterogeneity within the tumour which results in different sensitivities of tumour sub-populations to drug treatment, we are aware that tumour cells that are exposed to sub-lethal doses of chemotherapeutics are also likely to be distant from blood vessels, having limited access to nutrients and oxygen because of the poorly organized tumour vasculature [6,67]. We believe that future studies could involve conducting a similar siRNA screen under hypoxic conditions and comparing the list of hits with the data generated from the current study. It will also be of interest to examine whether the sensitization to cisplatin treatment observed here would be weakened, maintained, or enhanced under hypoxic and/or starvation conditions to determine whether the gene targets or pathways identified here should be further pursued from a pharmaceutical development perspective. Furthermore, potent inhibitors will be needed to successfully target the gene products as the new inhibitors may also encounter similar limitations to cisplatin in penetrating certain regions of the tumour. Drug delivery technologies may also be useful in increasing the availability of these agents for better drug distribution as well as in decreasing the rate of clearance [6,68,69]. Overall, the studies presented here highlight two important points: First, cancer cells react to cytotoxic drugs even when exposed to a dose that triggers no observable effect over 72hrs. This reaction involves multiple changes in gene expressions and activation of pathways such as those involved in chromatin modification and DNA repair. These results could help guide the development of targeted therapeutics to be used in combination with cisplatin to suppress survival responses induced within cancer cells when first exposed to sub-lethal concentrations of the drug. Second, pathways involved in INO80 chromatin remodeling and repairing of double strand breaks are up-regulated in response to low-dose cisplatin and should be targeted in chemotherapy-naive cells to improve the effectiveness of cisplatin treatment.

## Supporting Information

S1 Fig CABYR Knockdown sensitizes A549 and H460 cells to various concentrations of cisplatin. Cells were seeded and transfected as described in the Methods section for colony formation assays. Different concentrations of cisplatin were added the following day for 24 hours. The cells were then harvested, trypsinized, and seeded at 500 cells/well and incubated for 14 days undisturbed. The colonies were then stained with crystal violet and counted. The plating efficiency was calculated using the formula PE = # colonies formed/ #colonies plated. Data are averaged from all experimental trials and plotted as mean ± SEM. Statistical analyses were performed using two-way ANOVA followed by Sidak’s multiple comparisons test (*adjusted p-value <0.05). Our results show that CABYR knockdown alone has no effect on cell viability in A549 cells but enhances cisplatin activity significantly at low as well as high effect levels of cisplatin. In H460 cells, CABYR knockdown may have some effect of cell viability. The cisplatin-enhancing effect is observed at low to mid-doses of cisplatin. At 0.5 μM, sensitization was observed but the difference was not statistically significant. At 1 and 2 μM, the drug alone exerts a lethal effect that further sensitization was insignificant with CABYR silencing.
(TIF)

S2 Fig Differentially expressed genes are enriched in p53 signaling. Hierarchical clustering of the differentially expressed genes reveals pathway enrichment in p53 signaling, ascorbate and aldarate metabolism, and pentose and glucoronate interconversions.
(TIF)

S1 Table Primers Used for RT-PCR. (DOCX)

S2 Table GO Enrichment Analysis of Over-Expressed Genes Based on Biological Process Ontology. (DOCX)

S3 Table siRNA Screen Results for the Nucleotide Excision Repair Pathway. Genes that appeared to be cisplatin-potentiating targets according to our analysis criteria are highlighted in green. Genes that are highlighted in red are those that displayed lethality upon gene silencing via siRNA.
(DOCX)
