# Supplementary figures and images for: Combined Use of Gene Expression Modeling and siRNA Screening Identifies Genes and Pathways Which Enhance the Activity of Cisplatin When Added at No Effect Levels to Non-Small Cell Lung Cancer Cells In Vitro
Source: PLoS One. 2016 Mar 3;11(3):e0150675. doi: 10.1371/journal.pone.0150675 (PMC4777418; doi:10.1371/journal.pone.0150675)

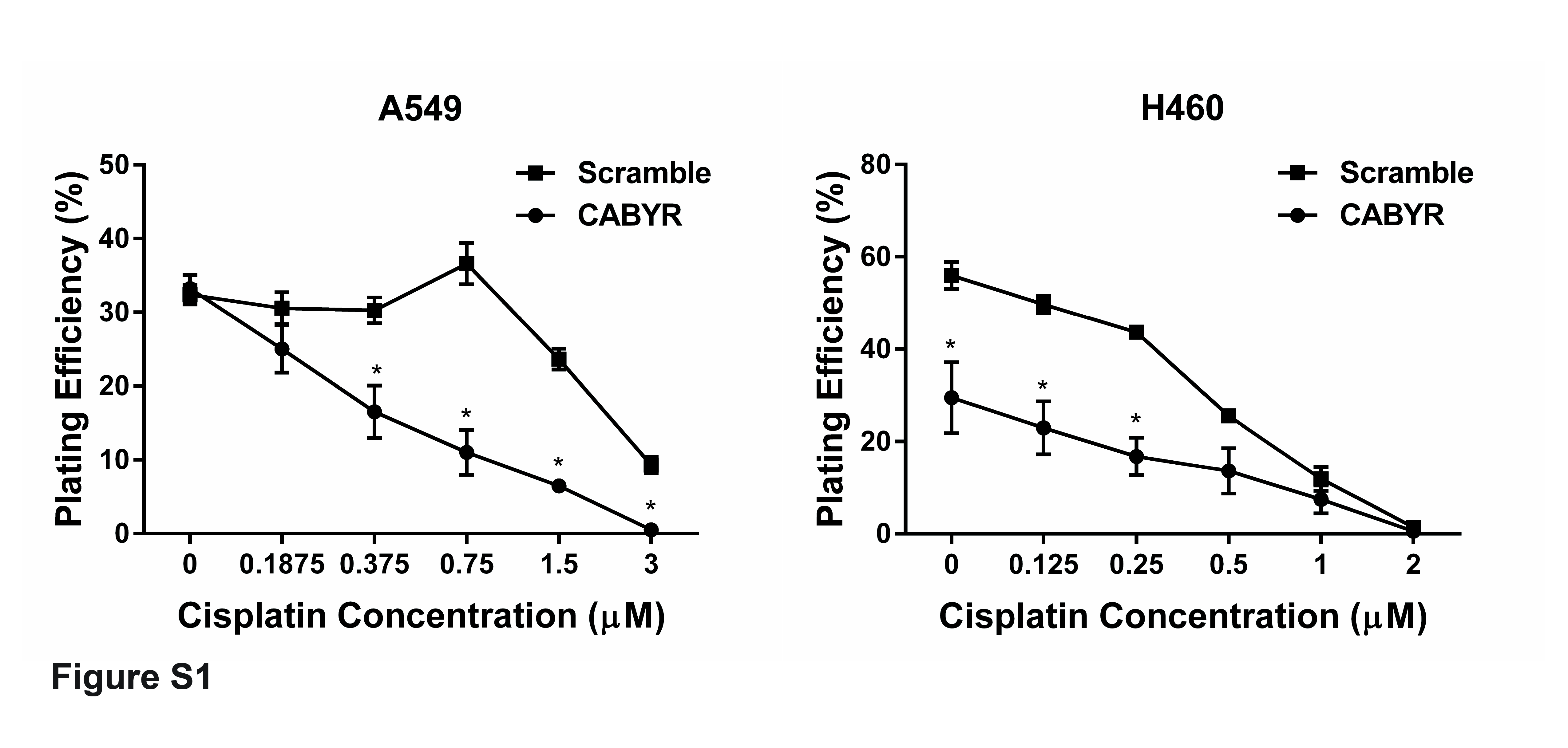

Supplement: S1 Fig — Cells were seeded and transfected as described in the Methods section for colony formation assays. Different concentrations of cisplatin were added the following day for 24 hours. The cells were then harvested, trypsinized, and seeded at 500 cells/well and incubated for 14 days undisturbed. The colonies were then stained with crystal violet and counted. The plating efficiency was calculated using the formula PE = # colonies formed/ #colonies plated. Data are averaged from all experimental trials and plotted as mean ± SEM. Statistical analyses were performed using two-way ANOVA followed by Sidak’s multiple comparisons test (*adjusted p-value <0.05). Our results show that CABYR knockdown alone has no effect on cell viability in A549 cells but enhances cisplatin activity significantly at low as well as high effect levels of cisplatin. In H460 cells, CABYR knockdown may have some effect of cell viability. The cisplatin-enhancing effect is observed at low to mid-doses of cisplatin. At 0.5 μM, sensitization was observed but the difference was not statistically significant. At 1 and 2 μM, the drug alone exerts a lethal effect that further sensitization was insignificant with CABYR silencing. (TIF) [file pone.0150675.s001.tif]

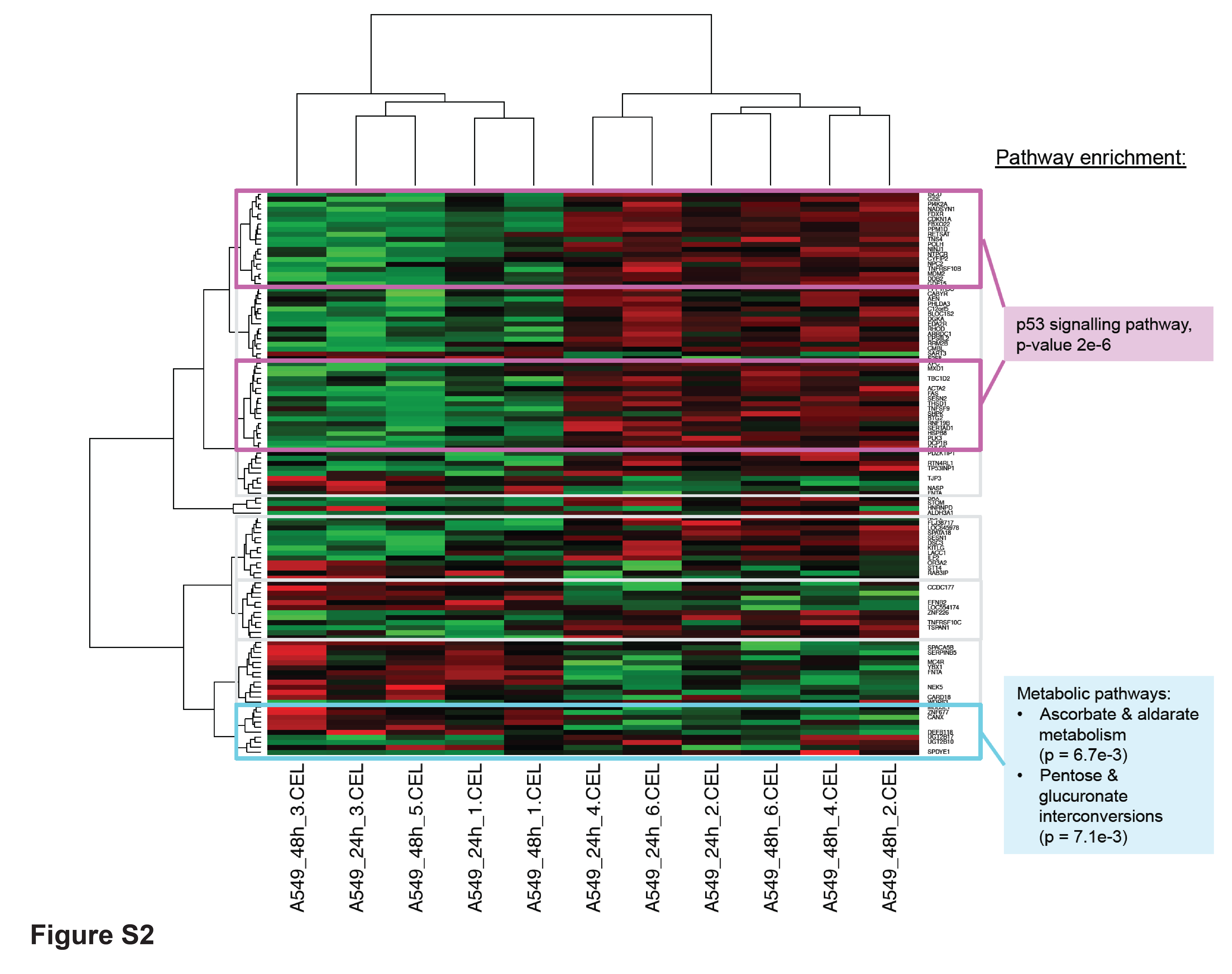

Supplement: S2 Fig — Hierarchical clustering of the differentially expressed genes reveals pathway enrichment in p53 signaling, ascorbate and aldarate metabolism, and pentose and glucoronate interconversions. (TIF) [file pone.0150675.s002.tif]
