# Supplementary material for: Combined Use of Gene Expression Modeling and siRNA Screening Identifies Genes and Pathways Which Enhance the Activity of Cisplatin When Added at No Effect Levels to Non-Small Cell Lung Cancer Cells In Vitro
Source: PLoS One. 2016 Mar 3;11(3):e0150675. doi: 10.1371/journal.pone.0150675 (PMC4777418; doi:10.1371/journal.pone.0150675)
Supplement: S1 Table — (DOCX) [file pone.0150675.s003.docx]

**S1 Table. Primers Used for RT-PCR**

| **Gene ID** | **TaqMan Assay ID** |
| --- | --- |
| ACTA2 | Hs00426835_g1 |
| ALDH3A1 | Hs00964880_m1 |
| CDKN1A | Hs00355782_m1 |
| DUSP4 | Hs01027785_m1 |
| EDA2R | Hs00939736_m1 |
| FAS | Hs00236330_m1 |
| FDXR | Hs01031617_m1 |
| FHL2 | Hs00179935_m1 |
| FNTA | Hs00357739_m1 |
| GDF15 | Hs00171132_m1 |
| NINJ1 | Hs00982607_m1 |
| RRM2B | Hs00968432_m1 |
| TP53INP1 | Hs00426835_g1 |
