# Supplementary material for: Combined Use of Gene Expression Modeling and siRNA Screening Identifies Genes and Pathways Which Enhance the Activity of Cisplatin When Added at No Effect Levels to Non-Small Cell Lung Cancer Cells In Vitro
Source: PLoS One. 2016 Mar 3;11(3):e0150675. doi: 10.1371/journal.pone.0150675 (PMC4777418; doi:10.1371/journal.pone.0150675)
Supplement: S2 Table — (DOCX) [file pone.0150675.s004.docx]

**S2 Table. GO Enrichment Analysis of Over-Expressed Genes Based on Biological Process Ontology**

| **GO biological process complete** | **Background Frequency** | **Sample frequency** | **Expected p-value** | **Fold Enrichment** | **+/-** | **P value^a^** |
| --- | --- | --- | --- | --- | --- | --- |
| intrinsic apoptotic signaling pathway in response to DNA damage by p53 class mediator | 30 | 5 | 0.12 | > 5 | + | 1.50E-03 |
| intrinsic apoptotic signaling pathway by p53 class mediator | 48 | 8 | 0.2 | > 5 | + | 2.75E-07 |
| negative regulation of fibroblast proliferation | 28 | 4 | 0.12 | > 5 | + | 4.84E-02 |
| cellular response to UV | 57 | 6 | 0.24 | > 5 | + | 1.25E-03 |
| intrinsic apoptotic signaling pathway in response to DNA damage | 79 | 7 | 0.33 | > 5 | + | 3.57E-04 |
| response to UV | 123 | 9 | 0.51 | > 5 | + | 2.04E-05 |
| signal transduction by p53 class mediator | 130 | 9 | 0.54 | > 5 | + | 3.28E-05 |
| cellular response to light stimulus | 97 | 6 | 0.4 | > 5 | + | 2.66E-02 |
| intrinsic apoptotic signaling pathway | 174 | 10 | 0.72 | > 5 | + | 2.52E-05 |
| apoptotic signaling pathway | 351 | 12 | 1.45 | > 5 | + | 1.93E-04 |
| response to light stimulus | 334 | 10 | 1.38 | > 5 | + | 1.00E-02 |
| response to radiation | 455 | 13 | 1.88 | > 5 | + | 3.88E-04 |
| cellular response to DNA damage stimulus | 692 | 16 | 2.86 | > 5 | + | 1.79E-04 |
| apoptotic process | 1054 | 20 | 4.35 | 4.59 | + | 6.64E-05 |
| cell death | 1117 | 21 | 4.62 | 4.55 | + | 3.06E-05 |
| death | 1122 | 21 | 4.64 | 4.53 | + | 3.31E-05 |
| programmed cell death | 1076 | 20 | 4.45 | 4.5 | + | 9.37E-05 |
| response to abiotic stimulus | 1065 | 17 | 4.4 | 3.86 | + | 1.15E-02 |
| cellular response to stress | 1630 | 24 | 6.73 | 3.56 | + | 2.15E-04 |
| regulation of apoptotic process | 1395 | 19 | 5.76 | 3.3 | + | 2.60E-02 |
| regulation of programmed cell death | 1412 | 19 | 5.83 | 3.26 | + | 3.10E-02 |
| response to stress | 3492 | 37 | 14.43 | 2.56 | + | 7.54E-05 |
| response to stimulus | 7510 | 56 | 31.03 | 1.8 | + | 3.28E-04 |

**^a^**The p-value is derived from the Pathway Studio software that determines the likelihood of the pathway being enriched from a random gene list of the same size.
