# Supplementary material for: Combined Use of Gene Expression Modeling and siRNA Screening Identifies Genes and Pathways Which Enhance the Activity of Cisplatin When Added at No Effect Levels to Non-Small Cell Lung Cancer Cells In Vitro
Source: PLoS One. 2016 Mar 3;11(3):e0150675. doi: 10.1371/journal.pone.0150675 (PMC4777418; doi:10.1371/journal.pone.0150675)
Supplement: S3 Table — Genes that appeared to be cisplatin-potentiating targets according to our analysis criteria are highlighted in green. Genes that are highlighted in red are those that displayed lethality upon gene silencing via siRNA. (DOCX) [file pone.0150675.s005.docx]

**S3 Table. siRNA Screen Results for the Nucleotide Excision Repair Pathway**

| **Gene Name** | **% Viability^a^ (Gene Knockdown + CDDP)** | **% Viability (Knockdown Only)** | **Gene Score Rank^b^** |
| --- | --- | --- | --- |
| CCNH | 82.13 | 100.00 | 9434 |
| CDK7 | 86.15 | 99.70 | 14219 |
| CETN2 | 63.64 | 74.58 | 17509 |
| CUL4A | 64.78 | 97.60 | 1744 |
| CUL4B | 43.66 | 58.41 | 16166 |
| DDB1 | 74.49 | 93.66 | 9335 |
| DDB2 | 62.56 | 93.72 | 3670 |
| ERCC1 | 34.03 | 45.29 | 18216 |
| ERCC2 | 65.29 | 92.25 | 2654 |
| ERCC3 | 73.12 | 94.75 | 7178 |
| ERCC4 | 38.87 | 75.86 | 3101 |
| ERCC5 | 67.20 | 82.03 | 12218 |
| ERCC6 | 40.50 | 60.64 | 14326 |
| LIG1 | 45.06 | 80.49 | 2493 |
| NMNAT1 | 51.82 | 79.36 | 7904 |
| PCNA | 42.79 | 63.75 | 12434 |
| RBX1 | 28.56 | 40.13 | 18769 |
| RFC1 | 52.01 | 91.13 | 1666 |
| RFC2 | 50.19 | 66.71 | 15275 |
| RFC3 | 54.56 | 101.85 | 851 |
| RFC4 | 60.99 | 82.92 | 9750 |
| RFC5 | 80.67 | 107.25 | 4893 |
| RPA1 | 81.14 | 101.09 | 8369 |
| RPA2 | 34.91 | 43.00 | 19536 |
| RPA3 | 78.56 | 98.56 | 8381 |
| XPA | 65.90 | 90.82 | 5791 |
| XPC | 72.92 | 95.88 | 7428 |

**^a^**Percent viability is normalized to transfection controls as described for the siRNA screen analysis in the Methods section.

^b^Gene score rank is the ranking based on the calculated gene score used to identify cisplatin-potentiating hits. The lower the value of the rank, the better the target is for enhancing cisplatin activity.
